# Melitracenium chloride

**DOI:** 10.1107/S1600536811022987

**Published:** 2011-06-18

**Authors:** Hoong-Kun Fun, Madhukar Hemamalini, M. S. Siddegowda, H. S. Yathirajan, B. Narayana

**Affiliations:** a X-ray Crystallography Unit, School of Physics, Universiti Sains Malaysia, 11800 USM, Penang, Malaysia; b Department of Studies in Chemistry, University of Mysore, Manasagangotri, Mysore 570 006, India; c Department of Studies in Chemistry, Mangalore University, Mangalagangotri 574 199, India,

## Abstract

In the title compound [systematic name: 3-(10,10-dimethyl­anthracen-9-yl­idene)-*N*,*N*,*N*-trimethyl­propanaminium chlor­ide], C_21_H_26_N^+^·Cl^−^, the cyclo­hexane ring adopts a chair conformation. The dihedral angle between the terminal benzene rings is 40.43 (12)°. In the crystal, ions are linked through inter­molecular N—H⋯Cl and C—H⋯Cl hydrogen bonds, forming supra­molecular layers parallel to the *bc* plane.

## Related literature

For the pharmaceutical properties of the title compound, see: Van Moffaert *et al.* (1983 ▶). For ring conformations, see: Cremer & Pople (1975 ▶).
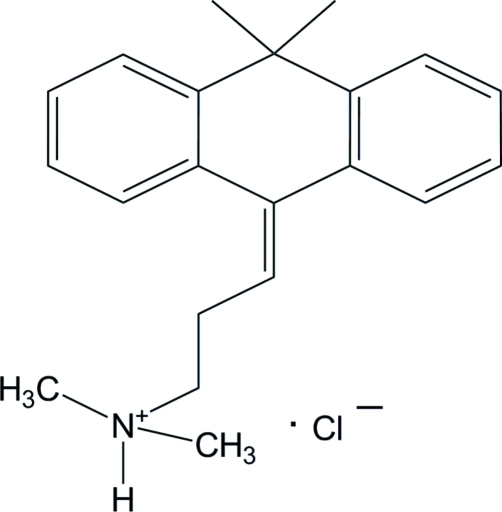


## Experimental

### 

#### Crystal data

C_21_H_26_N^+^·Cl^−^
*M* *_r_* = 327.88
Monoclinic, 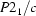

*a* = 15.0129 (18) Å
*b* = 8.8092 (11) Å
*c* = 14.0135 (17) Å
β = 91.506 (2)°
*V* = 1852.7 (4) Å^3^
*Z* = 4
Mo *K*α radiation
μ = 0.21 mm^−1^
*T* = 296 K
0.43 × 0.32 × 0.16 mm

#### Data collection

Bruker APEXII DUO CCD area-detector diffractometer
Absorption correction: multi-scan (*SADABS*; Bruker, 2009 ▶) *T*_min_ = 0.917, *T*_max_ = 0.967
19785 measured reflections
5366 independent reflections
3587 reflections with *I* > 2σ(*I*)
*R*_int_ = 0.039

#### Refinement

*R*[*F* ^2^ > 2σ(*F* ^2^)] = 0.063
*wR*(*F* ^2^) = 0.212
*S* = 1.05
5366 reflections
216 parameters
H atoms treated by a mixture of independent and constrained refinement
Δρ_max_ = 0.43 e Å^−3^
Δρ_min_ = −0.30 e Å^−3^

### 

Data collection: *APEX2* (Bruker, 2009 ▶); cell refinement: *SAINT* (Bruker, 2009 ▶); data reduction: *SAINT*; program(s) used to solve structure: *SHELXTL* (Sheldrick, 2008 ▶); program(s) used to refine structure: *SHELXTL*; molecular graphics: *SHELXTL*; software used to prepare material for publication: *SHELXTL* and *PLATON* (Spek, 2009 ▶).

## Supplementary Material

Crystal structure: contains datablock(s) global, I. DOI: 10.1107/S1600536811022987/rz2608sup1.cif

Structure factors: contains datablock(s) I. DOI: 10.1107/S1600536811022987/rz2608Isup2.hkl

Supplementary material file. DOI: 10.1107/S1600536811022987/rz2608Isup3.cml

Additional supplementary materials:  crystallographic information; 3D view; checkCIF report

## Figures and Tables

**Table 1:** Hydrogen-bond geometry (Å, °)

*D*—H⋯*A*	*D*—H	H⋯*A*	*D*⋯*A*	*D*—H⋯*A*
N1—H1*N*1⋯Cl1	0.98 (3)	2.03 (3)	3.0024 (19)	176 (3)
C17—H17*A*⋯Cl1^i^	0.97	2.81	3.710 (2)	155
C17—H17*B*⋯Cl1^ii^	0.97	2.77	3.717 (3)	164
C18—H18*B*⋯Cl1^iii^	0.96	2.68	3.625 (3)	169

Symmetry codes: (i) 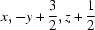; (ii) 
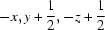
; (iii) 
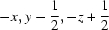
.

**Fig. 1.**
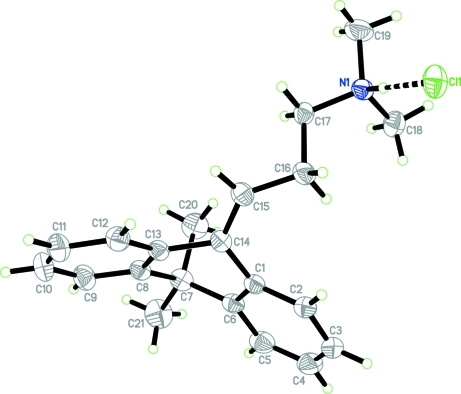
The asymmetric unit of the title compound, showing 50% probability displacement ellipsoids. The intramolecular N—H···Cl hydrogen bond is shown as a dashed line.

**Fig. 2.**
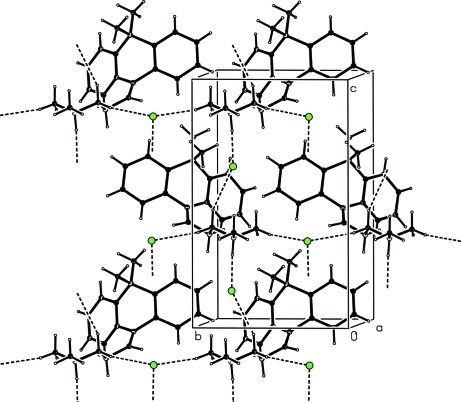
The crystal packing of the title compound. Hydrogen bonds are shown as dashed lines.

## supplementary crystallographic information

### Comment

Melitracen (systematic IUPAC name: 3-(10,10-dimethylanthracen-9(10*H*)-ylidene)-N,N-dimethylpropan-1-amine) is a tricyclic antidepressant (TCA) for the treatment of depression and anxiety. Its hydrochloride derivative has actions and effects similar to amitriptyline and is administered orally in the treatment of depression. Melitracen, a bipolar thymoleptic with activating properties in low dose, is usually coadministered with flupentixol in order to decrease the side effects. This combination has none serious side effects due to low drug dosage (Van Moffaert *et al.*, 1983). In view of the importance of the title compound, herein we report its crystal structure.

The asymmetric unit of the title compound (Fig 1) contains a melitracenium cation and a chloride anion. The central cyclohexane ring (C1/C6–C8/C13–C14) adopts a chair conformation with puckering parameters Q = 0.521 (2) Å, θ = 92.7 (2)° and φ = 298.8 (3)° (Cremer & Pople, 1975). The dihedral angle between the terminal benzene (C8–C13, C1–C6) rings is 40.43 (12)°. In the crystal structure (Fig. 2), the ions are linked through intermolecular N1—H1N1···Cl1, C17—H17A···Cl1, C17—H17B···Cl1 and C18—H18B···Cl1 (Table 1) hydrogen bonds, forming two-dimensional supramolecular layers parallel to the *bc* plane.

### Experimental

The title compound was obtained as a gift sample from R. L. Fine Chem. Ltd., Bangalore, India. The compound was recrystallized from methanol (m. p.: 512–514 K).

### Refinement

Atom H1N1 was located from a difference Fourier maps and refined freely [N–H = 0.97 (3) Å]. The remaining H atoms were positioned geometrically [C–H = 0.93–0.97 Å] and were refined using a riding model, with *U*_iso_(H) = 1.2 *U*_eq_(C). A rotating group model was applied to the methyl groups.

### Crystal data

**Table:**

C_21_H_26_N^+^·Cl^−^	*F*(000) = 704
*M**_r_* = 327.88	*D*_x_ = 1.176 Mg m^−^^3^
Monoclinic, *P*2_1_/*c*	Mo *K*α radiation, λ = 0.71073 Å
Hall symbol: -P 2ybc	Cell parameters from 4582 reflections
*a* = 15.0129 (18) Å	θ = 2.7–29.4°
*b* = 8.8092 (11) Å	µ = 0.21 mm^−^^1^
*c* = 14.0135 (17) Å	*T* = 296 K
β = 91.506 (2)°	Block, colourless
*V* = 1852.7 (4) Å^3^	0.43 × 0.32 × 0.16 mm
*Z* = 4	

### Data collection

**Table:**

Bruker APEXII DUO CCD area-detector diffractometer	5366 independent reflections
Radiation source: fine-focus sealed tube	3587 reflections with *I* > 2σ(*I*)
graphite	*R*_int_ = 0.039
φ and ω scans	θ_max_ = 30.0°, θ_min_ = 2.7°
Absorption correction: multi-scan (*SADABS*; Bruker, 2009)	*h* = −20→21
*T*_min_ = 0.917, *T*_max_ = 0.967	*k* = −12→12
19785 measured reflections	*l* = −19→19

### Refinement

**Table:**

Refinement on *F*^2^	Primary atom site location: structure-invariant direct methods
Least-squares matrix: full	Secondary atom site location: difference Fourier map
*R*[*F*^2^ > 2σ(*F*^2^)] = 0.063	Hydrogen site location: inferred from neighbouring sites
*wR*(*F*^2^) = 0.212	H atoms treated by a mixture of independent and constrained refinement
*S* = 1.05	*w* = 1/[σ^2^(*F*_o_^2^) + (0.0919*P*)^2^ + 0.9294*P*] where *P* = (*F*_o_^2^ + 2*F*_c_^2^)/3
5366 reflections	(Δ/σ)_max_ < 0.001
216 parameters	Δρ_max_ = 0.43 e Å^−^^3^
0 restraints	Δρ_min_ = −0.30 e Å^−^^3^

### Special details

**Table:**

Geometry. All s.u.'s (except the s.u. in the dihedral angle between two l.s. planes) are estimated using the full covariance matrix. The cell s.u.'s are taken into account individually in the estimation of s.u.'s in distances, angles and torsion angles; correlations between s.u.'s in cell parameters are only used when they are defined by crystal symmetry. An approximate (isotropic) treatment of cell s.u.'s is used for estimating s.u.'s involving l.s. planes.
Refinement. Refinement of F^2^ against ALL reflections. The weighted R-factor wR and goodness of fit S are based on F^2^, conventional R-factors R are based on F, with F set to zero for negative F^2^. The threshold expression of F^2^ > 2σ(F^2^) is used only for calculating R-factors(gt) etc. and is not relevant to the choice of reflections for refinement. R-factors based on F^2^ are statistically about twice as large as those based on F, and R- factors based on ALL data will be even larger.

### Fractional atomic coordinates and isotropic or equivalent isotropic displacement parameters (Å^2^)

**Table:**

	*x*	*y*	*z*	*U*_iso_*/*U*_eq_	
Cl1	0.03811 (5)	0.75554 (7)	0.14829 (4)	0.0618 (2)	
N1	0.03401 (12)	0.7409 (2)	0.36218 (13)	0.0440 (4)	
C1	0.34015 (14)	0.9607 (2)	0.49383 (15)	0.0431 (5)	
C2	0.37422 (16)	0.8711 (3)	0.42136 (19)	0.0564 (6)	
H2A	0.3513	0.8806	0.3593	0.068*	
C3	0.44190 (18)	0.7681 (3)	0.4410 (3)	0.0686 (8)	
H3A	0.4640	0.7085	0.3922	0.082*	
C4	0.4763 (2)	0.7535 (3)	0.5316 (3)	0.0738 (9)	
H4A	0.5213	0.6832	0.5446	0.089*	
C5	0.44455 (18)	0.8427 (3)	0.6034 (2)	0.0631 (7)	
H5A	0.4688	0.8324	0.6648	0.076*	
C6	0.37647 (14)	0.9487 (3)	0.58655 (17)	0.0466 (5)	
C7	0.33971 (16)	1.0491 (3)	0.66438 (16)	0.0505 (5)	
C8	0.32016 (14)	1.2066 (3)	0.62347 (15)	0.0434 (5)	
C9	0.33233 (19)	1.3385 (3)	0.67659 (19)	0.0610 (7)	
H9A	0.3545	1.3321	0.7390	0.073*	
C10	0.3118 (2)	1.4800 (3)	0.6378 (2)	0.0711 (8)	
H10A	0.3204	1.5671	0.6744	0.085*	
C11	0.2791 (2)	1.4919 (3)	0.5461 (2)	0.0672 (7)	
H11A	0.2661	1.5867	0.5200	0.081*	
C12	0.26541 (17)	1.3617 (3)	0.49243 (18)	0.0530 (6)	
H12A	0.2427	1.3697	0.4303	0.064*	
C13	0.28522 (14)	1.2188 (2)	0.53003 (15)	0.0412 (4)	
C14	0.27061 (14)	1.0775 (3)	0.47466 (14)	0.0416 (4)	
C15	0.19768 (16)	1.0598 (3)	0.41783 (15)	0.0480 (5)	
H15A	0.1633	1.1461	0.4068	0.058*	
C16	0.16583 (16)	0.9172 (3)	0.37049 (17)	0.0521 (6)	
H16A	0.2048	0.8334	0.3882	0.063*	
H16B	0.1662	0.9288	0.3017	0.063*	
C17	0.07204 (15)	0.8853 (3)	0.40238 (14)	0.0437 (5)	
H17A	0.0723	0.8797	0.4715	0.052*	
H17B	0.0338	0.9691	0.3830	0.052*	
C18	0.0842 (2)	0.6043 (3)	0.39637 (19)	0.0593 (6)	
H18A	0.1430	0.6057	0.3712	0.089*	
H18B	0.0536	0.5143	0.3751	0.089*	
H18C	0.0882	0.6050	0.4648	0.089*	
C19	−0.06121 (18)	0.7248 (4)	0.3865 (2)	0.0726 (8)	
H19A	−0.0846	0.6328	0.3591	0.109*	
H19B	−0.0943	0.8099	0.3616	0.109*	
H19C	−0.0663	0.7214	0.4546	0.109*	
C20	0.2502 (2)	0.9793 (4)	0.6960 (2)	0.0701 (8)	
H20A	0.2115	0.9649	0.6411	0.105*	
H20B	0.2224	1.0466	0.7401	0.105*	
H20C	0.2613	0.8832	0.7264	0.105*	
C21	0.4023 (2)	1.0579 (4)	0.7525 (2)	0.0798 (9)	
H21A	0.4602	1.0903	0.7336	0.120*	
H21B	0.4067	0.9596	0.7818	0.120*	
H21C	0.3790	1.1292	0.7972	0.120*	
H1N1	0.0369 (17)	0.750 (3)	0.293 (2)	0.048 (7)*	

### Atomic displacement parameters (Å^2^)

**Table:**

	*U*^11^	*U*^22^	*U*^33^	*U*^12^	*U*^13^	*U*^23^
Cl1	0.0908 (5)	0.0586 (4)	0.0356 (3)	0.0045 (3)	−0.0033 (3)	−0.0024 (2)
N1	0.0412 (9)	0.0561 (11)	0.0347 (8)	−0.0039 (8)	0.0010 (7)	−0.0039 (7)
C1	0.0384 (10)	0.0438 (11)	0.0471 (11)	−0.0067 (8)	0.0035 (8)	−0.0010 (8)
C2	0.0449 (13)	0.0620 (15)	0.0626 (14)	−0.0067 (11)	0.0066 (10)	−0.0133 (12)
C3	0.0469 (14)	0.0614 (17)	0.098 (2)	−0.0064 (12)	0.0139 (14)	−0.0238 (15)
C4	0.0483 (15)	0.0596 (17)	0.113 (3)	0.0060 (12)	−0.0039 (15)	−0.0030 (16)
C5	0.0510 (14)	0.0561 (15)	0.0815 (19)	−0.0003 (11)	−0.0097 (12)	0.0124 (13)
C6	0.0398 (11)	0.0457 (12)	0.0540 (12)	−0.0053 (9)	−0.0018 (9)	0.0084 (9)
C7	0.0543 (13)	0.0566 (14)	0.0406 (10)	−0.0045 (10)	−0.0013 (9)	0.0086 (9)
C8	0.0409 (11)	0.0497 (12)	0.0398 (10)	−0.0068 (9)	0.0022 (8)	−0.0004 (8)
C9	0.0677 (17)	0.0622 (16)	0.0528 (13)	−0.0090 (13)	−0.0024 (11)	−0.0129 (12)
C10	0.081 (2)	0.0486 (15)	0.084 (2)	−0.0099 (14)	−0.0014 (15)	−0.0177 (14)
C11	0.0720 (18)	0.0422 (13)	0.087 (2)	−0.0061 (12)	0.0023 (14)	0.0035 (13)
C12	0.0541 (14)	0.0474 (13)	0.0573 (13)	−0.0015 (10)	−0.0014 (10)	0.0073 (10)
C13	0.0395 (10)	0.0422 (11)	0.0419 (10)	−0.0059 (8)	0.0038 (8)	0.0012 (8)
C14	0.0452 (11)	0.0449 (11)	0.0346 (9)	−0.0042 (9)	0.0016 (8)	0.0016 (8)
C15	0.0508 (12)	0.0497 (12)	0.0431 (11)	−0.0040 (10)	−0.0045 (9)	−0.0006 (9)
C16	0.0478 (12)	0.0626 (15)	0.0458 (11)	−0.0053 (11)	−0.0017 (9)	−0.0115 (10)
C17	0.0483 (12)	0.0447 (11)	0.0382 (10)	0.0017 (9)	0.0014 (8)	−0.0039 (8)
C18	0.0683 (16)	0.0472 (13)	0.0628 (15)	0.0032 (12)	0.0074 (12)	−0.0010 (11)
C19	0.0431 (14)	0.095 (2)	0.0795 (19)	−0.0131 (14)	0.0083 (13)	−0.0070 (16)
C20	0.0776 (19)	0.0710 (18)	0.0628 (16)	−0.0100 (15)	0.0214 (13)	0.0165 (14)
C21	0.097 (2)	0.093 (2)	0.0487 (14)	0.0018 (19)	−0.0208 (14)	0.0090 (15)

### Geometric parameters (Å, °)

**Table:**

N1—C19	1.485 (3)	C11—C12	1.384 (4)
N1—C18	1.492 (3)	C11—H11A	0.9300
N1—C17	1.498 (3)	C12—C13	1.394 (3)
N1—H1N1	0.97 (3)	C12—H12A	0.9300
C1—C2	1.394 (3)	C13—C14	1.481 (3)
C1—C6	1.400 (3)	C14—C15	1.346 (3)
C1—C14	1.485 (3)	C15—C16	1.494 (3)
C2—C3	1.385 (4)	C15—H15A	0.9300
C2—H2A	0.9300	C16—C17	1.515 (3)
C3—C4	1.364 (5)	C16—H16A	0.9700
C3—H3A	0.9300	C16—H16B	0.9700
C4—C5	1.372 (5)	C17—H17A	0.9700
C4—H4A	0.9300	C17—H17B	0.9700
C5—C6	1.400 (4)	C18—H18A	0.9600
C5—H5A	0.9300	C18—H18B	0.9600
C6—C7	1.519 (4)	C18—H18C	0.9600
C7—C8	1.527 (3)	C19—H19A	0.9600
C7—C21	1.533 (3)	C19—H19B	0.9600
C7—C20	1.553 (4)	C19—H19C	0.9600
C8—C9	1.389 (3)	C20—H20A	0.9600
C8—C13	1.402 (3)	C20—H20B	0.9600
C9—C10	1.392 (4)	C20—H20C	0.9600
C9—H9A	0.9300	C21—H21A	0.9600
C10—C11	1.368 (5)	C21—H21B	0.9600
C10—H10A	0.9300	C21—H21C	0.9600
			
C19—N1—C18	109.3 (2)	C12—C13—C8	119.6 (2)
C19—N1—C17	110.8 (2)	C12—C13—C14	122.3 (2)
C18—N1—C17	112.33 (19)	C8—C13—C14	118.12 (19)
C19—N1—H1N1	107.7 (16)	C15—C14—C13	120.9 (2)
C18—N1—H1N1	110.5 (15)	C15—C14—C1	125.7 (2)
C17—N1—H1N1	106.2 (15)	C13—C14—C1	113.21 (18)
C2—C1—C6	119.5 (2)	C14—C15—C16	127.3 (2)
C2—C1—C14	122.0 (2)	C14—C15—H15A	116.4
C6—C1—C14	118.36 (19)	C16—C15—H15A	116.4
C3—C2—C1	120.5 (3)	C15—C16—C17	108.3 (2)
C3—C2—H2A	119.8	C15—C16—H16A	110.0
C1—C2—H2A	119.8	C17—C16—H16A	110.0
C4—C3—C2	120.4 (3)	C15—C16—H16B	110.0
C4—C3—H3A	119.8	C17—C16—H16B	110.0
C2—C3—H3A	119.8	H16A—C16—H16B	108.4
C3—C4—C5	119.9 (3)	N1—C17—C16	113.25 (18)
C3—C4—H4A	120.1	N1—C17—H17A	108.9
C5—C4—H4A	120.1	C16—C17—H17A	108.9
C4—C5—C6	121.6 (3)	N1—C17—H17B	108.9
C4—C5—H5A	119.2	C16—C17—H17B	108.9
C6—C5—H5A	119.2	H17A—C17—H17B	107.7
C1—C6—C5	118.1 (2)	N1—C18—H18A	109.5
C1—C6—C7	118.8 (2)	N1—C18—H18B	109.5
C5—C6—C7	123.0 (2)	H18A—C18—H18B	109.5
C6—C7—C8	109.23 (18)	N1—C18—H18C	109.5
C6—C7—C21	112.4 (2)	H18A—C18—H18C	109.5
C8—C7—C21	111.4 (2)	H18B—C18—H18C	109.5
C6—C7—C20	107.9 (2)	N1—C19—H19A	109.5
C8—C7—C20	107.9 (2)	N1—C19—H19B	109.5
C21—C7—C20	107.8 (2)	H19A—C19—H19B	109.5
C9—C8—C13	118.5 (2)	N1—C19—H19C	109.5
C9—C8—C7	122.5 (2)	H19A—C19—H19C	109.5
C13—C8—C7	119.0 (2)	H19B—C19—H19C	109.5
C8—C9—C10	121.0 (2)	C7—C20—H20A	109.5
C8—C9—H9A	119.5	C7—C20—H20B	109.5
C10—C9—H9A	119.5	H20A—C20—H20B	109.5
C11—C10—C9	120.4 (3)	C7—C20—H20C	109.5
C11—C10—H10A	119.8	H20A—C20—H20C	109.5
C9—C10—H10A	119.8	H20B—C20—H20C	109.5
C10—C11—C12	119.4 (3)	C7—C21—H21A	109.5
C10—C11—H11A	120.3	C7—C21—H21B	109.5
C12—C11—H11A	120.3	H21A—C21—H21B	109.5
C11—C12—C13	121.1 (2)	C7—C21—H21C	109.5
C11—C12—H12A	119.5	H21A—C21—H21C	109.5
C13—C12—H12A	119.5	H21B—C21—H21C	109.5
			
C6—C1—C2—C3	1.6 (4)	C7—C8—C9—C10	−178.4 (3)
C14—C1—C2—C3	177.0 (2)	C8—C9—C10—C11	0.0 (5)
C1—C2—C3—C4	−0.3 (4)	C9—C10—C11—C12	0.8 (5)
C2—C3—C4—C5	−0.8 (5)	C10—C11—C12—C13	−0.6 (4)
C3—C4—C5—C6	0.5 (5)	C11—C12—C13—C8	−0.5 (4)
C2—C1—C6—C5	−1.9 (3)	C11—C12—C13—C14	179.5 (2)
C14—C1—C6—C5	−177.4 (2)	C9—C8—C13—C12	1.3 (3)
C2—C1—C6—C7	178.9 (2)	C7—C8—C13—C12	178.7 (2)
C14—C1—C6—C7	3.4 (3)	C9—C8—C13—C14	−178.7 (2)
C4—C5—C6—C1	0.8 (4)	C7—C8—C13—C14	−1.2 (3)
C4—C5—C6—C7	180.0 (3)	C12—C13—C14—C15	−39.9 (3)
C1—C6—C7—C8	−39.0 (3)	C8—C13—C14—C15	140.1 (2)
C5—C6—C7—C8	141.8 (2)	C12—C13—C14—C1	143.6 (2)
C1—C6—C7—C21	−163.2 (2)	C8—C13—C14—C1	−36.4 (3)
C5—C6—C7—C21	17.7 (3)	C2—C1—C14—C15	43.7 (3)
C1—C6—C7—C20	78.1 (3)	C6—C1—C14—C15	−140.9 (2)
C5—C6—C7—C20	−101.1 (3)	C2—C1—C14—C13	−140.0 (2)
C6—C7—C8—C9	−144.6 (2)	C6—C1—C14—C13	35.5 (3)
C21—C7—C8—C9	−19.9 (3)	C13—C14—C15—C16	−169.1 (2)
C20—C7—C8—C9	98.3 (3)	C1—C14—C15—C16	6.9 (4)
C6—C7—C8—C13	38.0 (3)	C14—C15—C16—C17	122.4 (3)
C21—C7—C8—C13	162.7 (2)	C19—N1—C17—C16	−173.4 (2)
C20—C7—C8—C13	−79.1 (3)	C18—N1—C17—C16	64.1 (2)
C13—C8—C9—C10	−1.0 (4)	C15—C16—C17—N1	−177.84 (18)

### Hydrogen-bond geometry (Å, °)

**Table:**

*D*—H···*A*	*D*—H	H···*A*	*D*···*A*	*D*—H···*A*
N1—H1N1···Cl1	0.98 (3)	2.03 (3)	3.0024 (19)	176 (3)
C17—H17A···Cl1^i^	0.97	2.81	3.710 (2)	155
C17—H17B···Cl1^ii^	0.97	2.77	3.717 (3)	164
C18—H18B···Cl1^iii^	0.96	2.68	3.625 (3)	169

Symmetry codes: (i) *x*, −*y*+3/2, *z*+1/2; (ii) −*x*, *y*+1/2, −*z*+1/2; (iii) −*x*, *y*−1/2, −*z*+1/2.
